# Supplementary material for: Convergent Evolution of Armor: Thermal Resistance in Deep-Sea Hydrothermal Vent Crustaceans
Source: Biology (Basel). 2024 Nov 21;13(12):956. doi: 10.3390/biology13120956 (PMC11673863; doi:10.3390/biology13120956)
Supplement: Supplementary file 1 [file biology-13-00956-s001.zip › biology-3285576-supplementary.pdf]

1. Supplementary Materials

1.1 PCR

PCR was conducted using following primer sets: Purple hermit crab LCO1490/HCO2198 for the mtCOI gene [71]; 16Sar/16Sbr for the mt16S rRNA gene [72]; 18S1F/18S5R for the 18S rRNA gene [73]; H3F/H3R for the H3 gene [74]. PCR mixture was prepared in 20 µl contained 10 µl of IP pro-Taq PCR Master mix (Cosmogenetech, Seoul, Korea) and 1 µl of each forward and reverse primer (10 pmol/µl), 1 µl of genomic DNA (20-100ng), and distilled water up to the final volume. Thermocycling was carried out with the following cycles: 5 min at 94 °C for initial denaturation, followed by 35 cycles at 94 °C for 30 s, 45-50 °C for 30 s (45°C for COI and 50 °C for the other three genes),72 °C for 30 s, and finally, 7 min at 72°C for final extension.

1.2 Molecular analysis

Species of newly added specimens in the present study were identified based on mtCOI sequences using the BLAST algorithm against the GenBank nucleotide collection (nr/nt) database. Every sequence had a pairwise sequence similarity of at least 98% to reference sequences.

For phylogenetic analysis, each sequence of four genes (Table S1) aligned for each gene using the MUSCLE algorithm v. 3.8.425 [75] in Geneious Prime 2022.2. and overlapping fragments were then used for the subsequent steps. Poorly alignment regions of ribosomal RNA fragments were removed using Gblock 0.91b [76] with default settings. Process for assessing the phylogenetic relationship was conducted based on the Baysian ingerence (BI) and Maximum likelihood (ML) approaches on the CIPRES web server [36]. Aligned fragments of four genes were concatenated into a single sequence per species. The best fitting model of nucleotide substitution for each gene was estimated using JModelTest2 [77] based on the Akaike Information Creterion (AIC). The ML analysis was conducted using IQ-Tree v. 2.2.2.7 [34,35]

with 100,000 ultrafast bootstraps. The BI was conducted using MrBayes 3.2.7 [32,33] based on Metropolis coupled Monte Carlo Markov Chains (MCMC) length of 100 million generations with four chains and sampling every 1000 generations after the burn-in of the first 25%.

2. Supplementary Figures and Tables

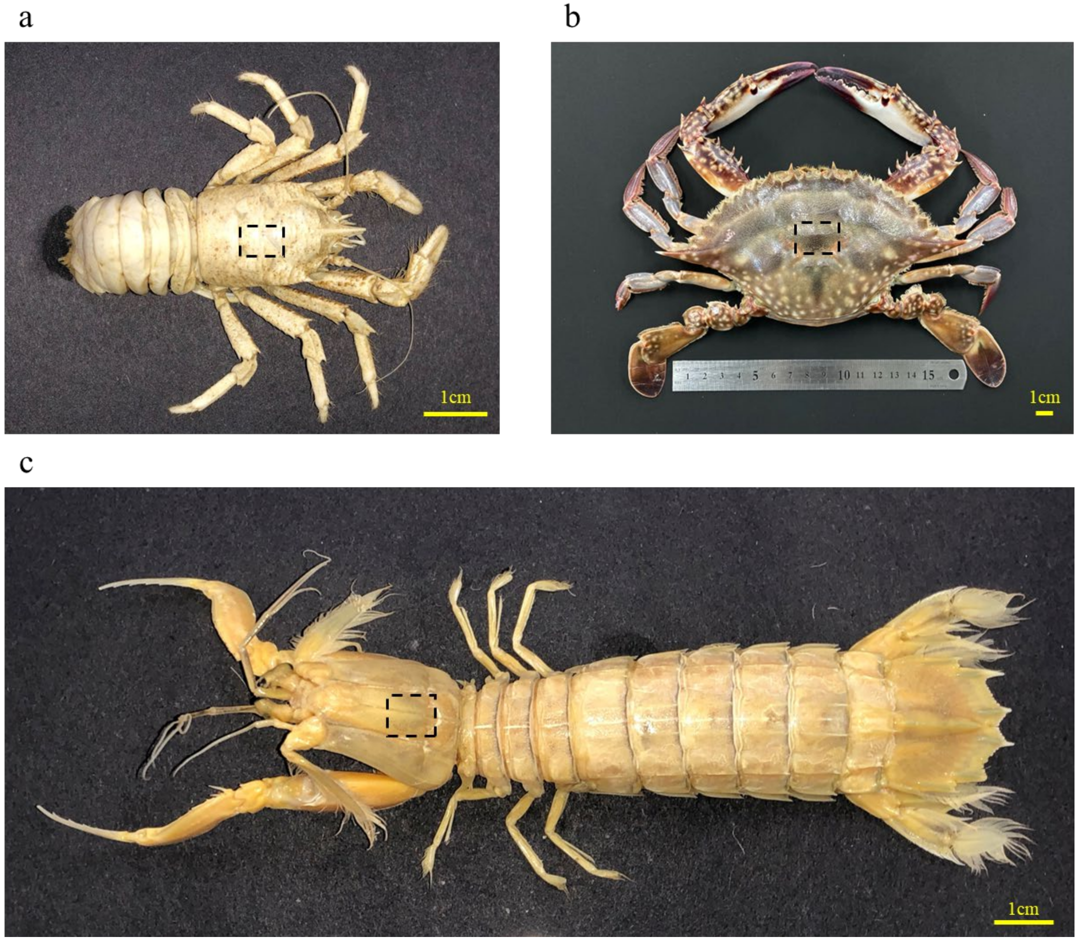

Supplementary Figure S1. Crustacean sample image (a, vent squat lobster; b, blue crab; c, mantis shrimp). The square with dotted line represents the part used for analysis.

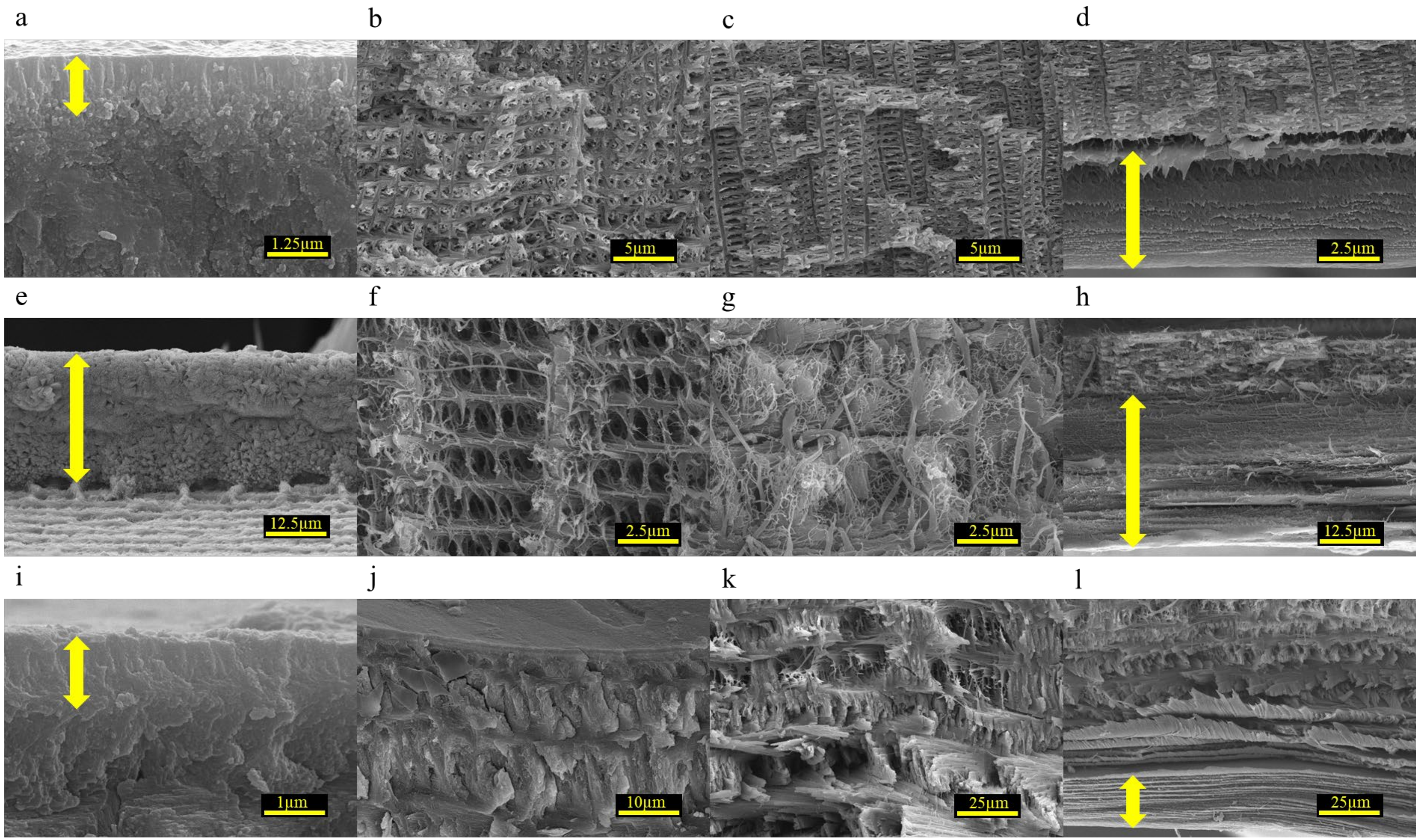

Supplementary Figure S2. Microstructure of each layer of the VSL (a-d), Blue crab (e-h), Mantis shrimp (i-l). Epicuticle (a, e, i), exocuticle (b, f, j), endocuticle (c, g, k), and membrane layer (d, h, l). Yellow arrows indicate epicuticle (a, e, i) and membrane layer (d, h, l).

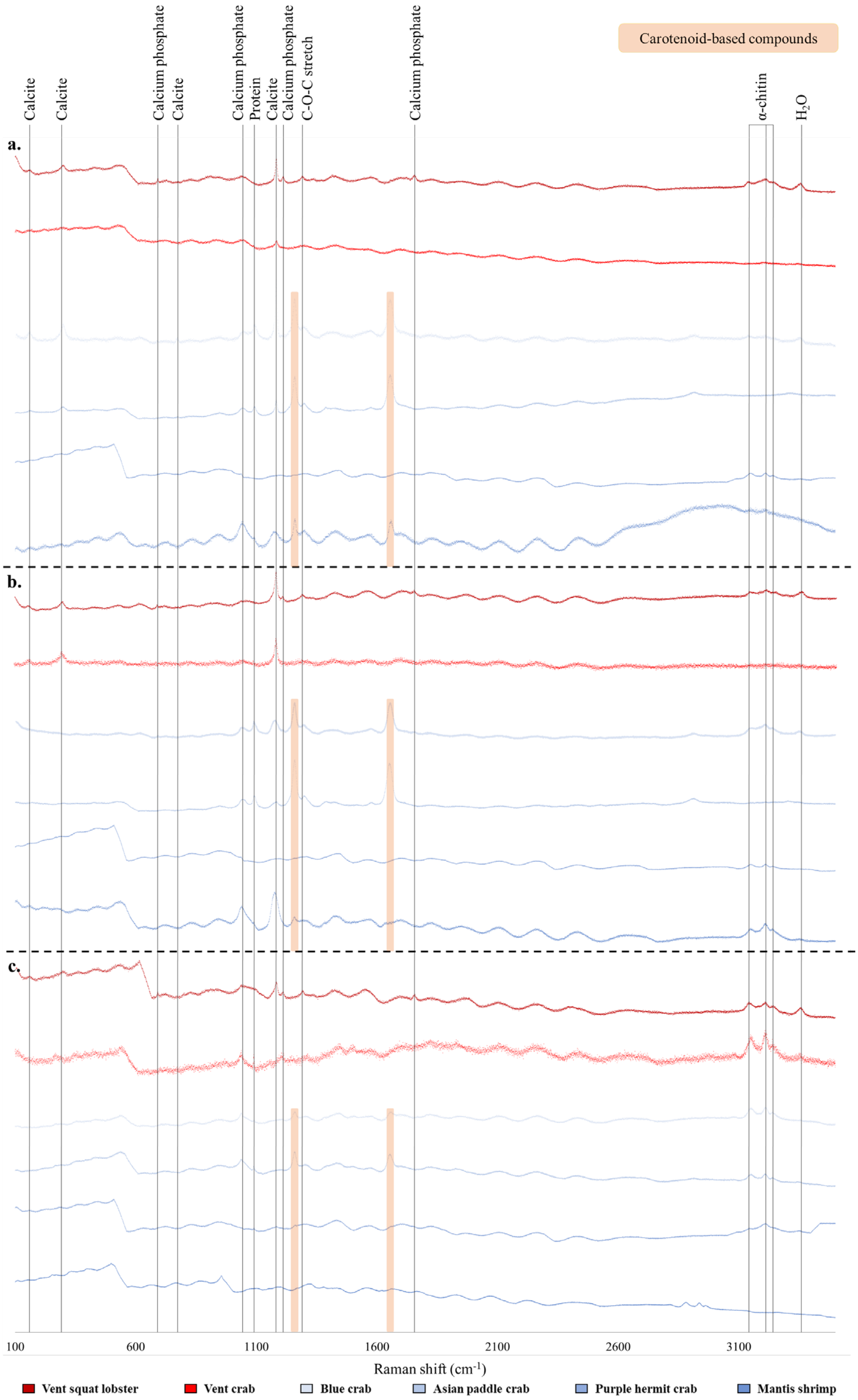

8

Supplementary Figure S3. Raman analysis graph of epicuticle (a), exocuticle (b), and membrane layer (c). 9

Supplementary Table S1. GenBank accession IDs associated to phylogenetic analysis in this study.

Specimen	Species	COI	16S rRNA	18S rRNA	H3
Blue crab	*Portunus trituberculatus*	OQ644533	OQ629572	OQ629561	OQ629550
Asian paddle crab	*Charybdis japonica*	OQ644536	OQ629575	OQ629562	OQ629551
Vent crab	*Austinograea* sp.	OQ644540	OQ629579	OQ629565	OQ629554
Vent squat lobster	*Munidopsis lauensis*	OQ644542	OQ629581	OQ629566	OQ629555
Purple hermit crab	*Elassochirus cavimanus*	OR462714	OR467411	OR467414	OR508504
Mantis shrimp	*Oratosquilla oratoria*	OR462784	OR467412	OR467415	OR508505

Supplementary Table S2. Substitution model for each gene used for ML and BI analysis.

Gene	Maximum Likelihood tree	Bayesian Inference tree
mtCOI	GTR + I + Γ	GTR + I + Γ
mt16S rRNA	TVM + I	GTR + I
18S rRNA	TrN + I + Γ	GTR + I + Γ
H3	GTR + G	GTR + Γ

Supplementary Table S3. Contents of the elements constituting each layer of the crustacean exoskeleton (mean ± SE).

Layers	Species	C	N	O	Fe	Na	Mg	Al	Si	P	Zr	S	Cl	Ca
Epicuticle	Vent squat lobster	58.6±4.93	6.79±0.69	23.05±3.13	-	0.23±0.04	0.52±0.1	1.6±0.09	0.08±0.02	0.04±0.02	0.66±0.09	0.29±0.05	0.67±0.29	7.46±1.5
	Vent crab	65.86±1.36	9.23±0.38	14.62±2.12	-	0.1±0.03	0.18±0.04	1.91±0.42	0.09±0.04	0.01±0.01	1.2±0.42	0.9±0.18	3.11±0.76	2.8±1.68
	Blue crab	43.17±2.23	8.79±1.8	35.65±3.07	0.32±0	0.46±0.04	0.7±0.08	1.08±0.29	0.16±0.03	0.01±0.01	0.8±0.16	0.17±0.07	0.27±0.06	8.42±1.76
	Asian paddle crab	37.8±1.14	5.38±0.79	42.57±2.93	-	0.55±0.11	0.92±0.06	0.47±0.18	0.43±0.18	0.03±0.02	0.68±0.2	0.12±0.04	0.21±0.06	10.85±1.76
	Purple hermit crab	60.1±2.99	6.69±1.92	11.38±2.51	0.62±0.2	0.09±0.03	0.48±0.08	5.47±1.31	1.22±0.68	-	2.47±0.72	0.49±0.14	2.41±0.94	8.56±2.23
	Mantis shrimp	19.02±0.86	6.44±0.2	42.7±0.69	0.39±0.06	0.23±0.05	2.7±0.23	0.35±0.32	0.12±0.03	6.26±0.61	1.46±0.2	0.28±0.01	-	20.04±0.27
Exocuticle	Vent squat lobster	21.49±0.57	2.79±0.09	48.98±1.04	-	0.9±0.08	1.27±0.12	0.21±0.09	0.22±0.09	0.27±0.07	0.54±0.08	0.41±0.09	0.14±0.07	22.79±0.66
	Vent crab	19.05±1.75	2.81±0.53	50.43±0.97	-	0.74±0.05	2.56±0.07	0.15±0.05	0.15±0.07	0.55±0.11	0.82±0.08	0.17±0.06	0.12±0.05	22.43±1.58
	Blue crab	23.39±2.37	4.94±0.08	35.27±1.32	1.14±0.1	0.83±0.01	2.37±0.19	0.7±0.01	0.66±0.02	4.68±0.22	1.61±0.06	0.65±0.03	0.85±0.06	22.92±0.6
	Asian paddle crab	16.63±0.68	2.69±0.02	48.16±2.58	-	0.55±0.04	3.01±0.07	0.17±0.14	0.26±0.12	1.23±0.19	0.92±0.08	0.3±0.15	0.2±0.09	25.86±2.71
	Purple hermit crab	57.04±0.72	11.99±0.86	23.1±0.22	0.18±0.01	0.06±0.01	0.32±0.03	1.94±0.48	0.75±0.34	0.46±0.1	1.71±0.24	0.03±0.01	0.01±0.01	2.42±0.34
	Mantis shrimp	11.04±0.28	6.25±0.33	30.52±1.1	0.78±0.06	0.77±0.05	2.56±0.05	0.97±0.19	0.83±0.1	3.93±0.36	1.67±0.03	0.8±0.05	0.21±0.04	39.68±1.52
Endocuticle	Vent squat lobster	32.47±1.1	3.69±0.09	42.05±1.16	-	0.68±0.04	1.82±0.03	0.18±0.06	0.22±0.05	0.9±0.11	0.65±0.1	0.31±0.08	0.16±0.04	16.9±0.95
	Vent crab	18.12±0.5	2.71±0.04	48.77±1.14	-	0.83±0.08	2.24±0.14	0.25±0.11	0.34±0.1	1.28±0.19	0.85±0.11	0.32±0.07	0.16±0.08	24.13±0.9
	Blue crab	34.53±1.8	5.9±0.3	32.57±3.64	0.53±0.02	0.66±0.02	1.32±0.16	0.75±0.12	0.45±0.1	1.41±0.1	1.2±0.08	0.43±0.08	0.44±0.07	19.81±1.48
	Asian paddle crab	18.52±0.5	3.44±0.59	44.99±3.67	-	0.71±0.03	1.78±0.13	0.36±0.15	0.43±0.14	1.32±0.16	1.14±0.24	0.38±0.11	0.22±0.08	26.71±1.71
	Purple hermit crab	65.72±0.38	11.94±1.33	18.85±0.1	0.15±0.01	0.1±0.03	0.13±0.04	0.59±0.39	-	0.01±0.01	1.19±0.2	-	0.01±0.01	1.03±0.27
	Mantis shrimp	42.29±1.46	8.56±1.1	17.52±2.18	1.15±0.13	0.24±0.06	1.37±0.17	1.2±0.82	1.03±0.8	2.72±0.13	2.01±0.28	0.31±0.09	0.08±0.04	21.51±1.08
Membrane	Vent squat lobster	71.5±3.62	7.58±1.17	18.42±3.08	-	0.08±0.02	0.13±0.05	0.16±0.07	0.11±0.05	0.18±0.11	0.64±0.18	0.25±0.12	0.16±0.03	0.79±0.2
	Vent crab	60.09±1.01	7.04±2.18	20.84±3.15	-	0.15±0.01	0.59±0.3	0.16±0.07	0.48±0.13	1.54±1.51	1.55±0.76	0.39±0.16	0.43±0.18	6.73±3.25
	Blue crab	65.19±1.6	9.39±0.92	23.15±1.36	0.25±0.06	0.12±0.03	0.05±0.01	0.19±0.02	0.1±0.02	0.01±0.01	0.69±0.15	0.24±0.06	0.35±0.14	0.26±0.05
	Asian paddle crab	56.31±5.06	7.34±1.3	21.63±1	-	0.11±0.04	0.65±0.3	0.54±0.22	0.41±0.25	3.09±1.58	1.44±0.39	0.36±0.13	0.79±0.56	7.33±4.38
	Purple hermit crab	-	-	-	-	-	-	-	-	-	-	-	-	-
	Mantis shrimp	63.89±0.98	14.29±0.54	20.29±0.54	0.18±0.02	0.07±0.01	0.09±0	0.26±0.15	0.08±0.05	-	0.61±0.03	0.03±0.01	-	0.21±0.04

Supplementary Table S4. Statistical results of comparative analysis of the elements of each layer of the exoskeleton of six species of crustaceans.

Layers	Epicuticle		Exocuticle		Endocuticle		Membrane	
Elements	F or χ^2	p	F or χ^2	p	F or χ^2	p	F or χ^2	p
C	$F_{5,18} = 19.379$	< 0.001	$\chi^2 = 15.513$	0.008	$F_{5,18} = 202.511$	< 0.001	$F_{4.15} = 3.675$	0.039
N	-	-	$\chi^2 = 15.555$	0.008	$\chi^2 = 16.768$	0.005	$F_{4.15} = 4.987$	0.015
O	$F_{5,18} = 16.664$	< 0.001	$F_{5,18} = 66.794$	< 0.001	$F_{5,18} = 33.656$	< 0.001	-	-
Fe	-	-	-	-	-	-	-	-
Na	$F_{5,18} = 10.401$	< 0.001	$F_{5,18} = 12.786$	< 0.001	$F_{5,18} = 35.124$	< 0.001	-	-
Mg	$\chi^2 = 16.797$	0.005	$F_{5,18} = 5.941$	0.004	$F_{5,18} = 8.737$	< 0.001	-	-
Al	$\chi^2 = 12.710$	0.026	$\chi^2 = 12.611$	0.027	-	-	-	-
Si	-	-	$\chi^2 = 13.555$	0.019	-	-	-	-
P	$\chi^2 = 13.228$	0.021	$F_{5,18} = 68.167$	< 0.001	$F_{5,18} = 18.947$	< 0.001	-	-
Zr	$F_{5,18} = 3.816$	0.024	$F_{5,18} = 79.008$	< 0.001	$F_{5,18} = 15.941$	< 0.001	-	-
S	$F_{5,18} = 4.029$	0.02	$F_{5,18} = 12.417$	< 0.001	-	-	-	-
Cl	$\chi^2 = 15.824$	0.007	$\chi^2 = 13.218$	0.021	-	-	-	-
Ca	$F_{5,18} = 23.032$	< 0.001	$F_{5,18} = 77.961$	< 0.001	$F_{5,18} = 65.601$	< 0.001	-	-

Supplementary Table S5. Raman data / cm-1 of five species of the crustacean exoskeleton.

Raman shift (cm^{-1})	Substances	References
151-154, 281-283, 710-713	Calcite, amorphous calcium carbonate (ACC)	[78–80]
638-639, 954-956, 1111-1113, 1607-1610	Phosphate	[62,81]
1001-1002	Ring breathing, protein	[82–85]
1086-1090	Calcite, ACC	[62,78–80,86]
1154-1160	ATX, α-chitin, crustacyanin	[62,87–89]
1184	C-O-C stretch	[90]
1513-1517	ATX, β-carotene, related carotenoids	[87,88]
2880-3020	A-chitin	[87]
3067-3071	H_2O	[78,87]

Supplementary Table S6. Supplementary Table S6. Weight loss (%) from TGA (mean ± SE).

Range \ Species	Vent squat lobster	Vent crab	Blue crab	Asian paddle crab	Purple hermit crab	Mantis shrimp	Vent	Coastal
**1**	4.24 ± 0.24	3.88 ± 0.24	13.48 ± 0.12	9.54 ± 0.64	8.72 ± 0.57	13.63 ± 0.41	4.06 ± 0.41	11.34 ± 2.37
**2**	13.91 ± 0.52	12.07 ± 0.59	18.96 ± 0.19	15.34 ± 2.49	28.5 ± 1.96	24.01 ± 3.53	12.99 ± 1.28	21.7 ± 5.59
**3**	35.15 ± 0.22	32.27 ± 1.03	25.03 ± 1.14	27.67 ± 1.77	21.01 ± 1.14	15.58 ± 2.14	33.71 ± 1.68	22.32 ± 4.95
